# Phenyl *N*-[4-chloro-3-(trifluoro­meth­yl)phen­yl]carbamate

**DOI:** 10.1107/S1600536809004942

**Published:** 2009-02-18

**Authors:** Hai-Tao Tang, Zheng Fang

**Affiliations:** a College of Life Sciences and Pharmaceutical Engineering, Nanjing University of Technology, Xinmofan Road No.5 Nanjing, Nanjing 210009, People’s Republic of China

## Abstract

In the mol­ecule of the title compound, C_14_H_9_ClF_3_NO_2_, the aromatic rings are oriented at a dihedral angle of 66.49 (3)°. Intra­molecular C—H⋯F and C—H⋯O inter­actions result in the formation of one planar five- and one non-planar six-membered ring. In the crystal structure, inter­molecular N—H⋯O hydrogen bonds link the mol­ecules into chains.

## Related literature

For bond-length data, see: Allen *et al.* (1987 ▶).
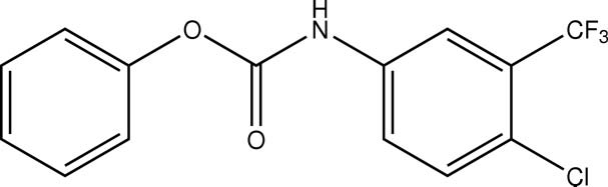


## Experimental

### 

#### Crystal data

C_14_H_9_ClF_3_NO_2_
*M* *_r_* = 315.67
Orthorhombic, 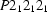

*a* = 8.5680 (17) Å
*b* = 11.152 (2) Å
*c* = 14.232 (3) Å
*V* = 1359.9 (5) Å^3^
*Z* = 4
Mo *K*α radiation
μ = 0.32 mm^−1^
*T* = 294 K
0.30 × 0.20 × 0.10 mm

#### Data collection

Enraf–Nonius CAD-4 diffractometer
Absorption correction: ψ scan (North *et al.*, 1968 ▶) *T*_min_ = 0.910, *T*_max_ = 0.969
2733 measured reflections
2465 independent reflections
1775 reflections with *I* > 2σ(*I*)
*R*_int_ = 0.034
3 standard reflections frequency: 120 min intensity decay: 1%

#### Refinement

*R*[*F* ^2^ > 2σ(*F* ^2^)] = 0.066
*wR*(*F* ^2^) = 0.188
*S* = 1.00
2465 reflections
190 parameters
H-atom parameters constrained
Δρ_max_ = 0.31 e Å^−3^
Δρ_min_ = −0.33 e Å^−3^
Absolute structure: Flack (1983 ▶), 1012 Friedel pairs
Flack parameter: −0.1 (2)

### 

Data collection: *CAD-4 Software* (Enraf–Nonius, 1989 ▶); cell refinement: *CAD-4 Software*; data reduction: *XCAD4* (Harms & Wocadlo, 1995 ▶); program(s) used to solve structure: *SHELXS97* (Sheldrick, 2008 ▶); program(s) used to refine structure: *SHELXL97* (Sheldrick, 2008 ▶); molecular graphics: *PLATON* (Spek, 2009 ▶); software used to prepare material for publication: *SHELXL97*.

## Supplementary Material

Crystal structure: contains datablocks global, I. DOI: 10.1107/S1600536809004942/hk2623sup1.cif

Structure factors: contains datablocks I. DOI: 10.1107/S1600536809004942/hk2623Isup2.hkl

Additional supplementary materials:  crystallographic information; 3D view; checkCIF report

## Figures and Tables

**Table 1:** Hydrogen-bond geometry (Å, °)

*D*—H⋯*A*	*D*—H	H⋯*A*	*D*⋯*A*	*D*—H⋯*A*
N—H0*A*⋯O2^i^	0.86	2.10	2.943 (5)	168
C9—H9*A*⋯O2	0.93	2.44	2.950 (5)	114
C13—H13*A*⋯F2	0.93	2.34	2.687 (5)	102

Symmetry code: (i) 
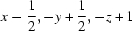
.

**Fig. 1.**
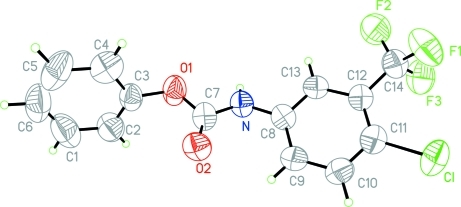
The molecular structure of the title molecule, with the atom-numbering scheme. Displacement ellipsoids are drawn at the 30% probability level.

**Fig. 2.**
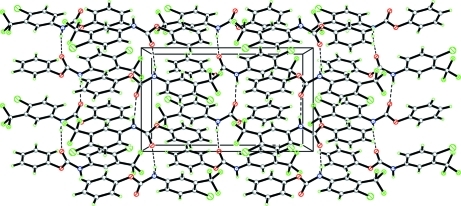
A partial packing diagram of the title compound. Hydrogen bonds are shown as dashed lines.

## supplementary crystallographic information

### Comment

Some derivatives of benzoic acid are important chemical materials. We report herein the crystal structure of the title compound.

In the molecule of the title compound (Fig. 1), the bond lengths (Allen *et al.*,  1987) and angles are within normal ranges. Rings A (C1-C6) and B (C8-C13) are, of course, planar and the dihedral angle between them is A/B = 66.49 (3)°. The intramolecular C-H···F and C-H···O interactions (Table 1) result in the formations of one planar five- and one nonplanar six-membered rings C (F2/C12-C14/H13A) and D (O2/N/C7-C9/H9A). Ring C is oriented with respect to rings A and B at dihedral angles of 66.33 (3)° and 0.93 (3)°, respectively. So, rings B and C are nearly coplanar.

In the crystal structure, intermolecular N-H···O hydrogen bonds (Table 1) link the molecules into chains (Fig. 2), in which they may be effective in the stabilization of the structure.

### Experimental

For the preparation of the title compound, phenyl chloroformate (1.0 ml) was added slowly to a cold solution of 4-chloro-3-(trifluoromethyl)benzenamine (1.0 g) and triethylamine (0.8 ml) in methylene chloride (10 ml) at 273 K. The mixture was then warmed and stirred for 1 h at room temperature. Then, it was washed with water (20 ml), dried and concentrated to give the title compound (yield; 1.3 g). Crystals suitable for X-ray analysis were obtained by slow evaporation of an methanol solution.

### Refinement

H-atoms were positioned geometrically, with N-H = 0.86 Å (for NH) and C-H = 0.93 Å for aromatic H, and constrained to ride on their parent atoms, with U_iso_(H) = 1.2U_eq_(C, N).

### Crystal data

**Table:**

C_14_H_9_ClF_3_NO_2_	*F*(000) = 640
*M**_r_* = 315.67	*D*_x_ = 1.542 Mg m^−^^3^
Orthorhombic, *P*2_1_2_1_2_1_	Mo *K*α radiation, λ = 0.71073 Å
Hall symbol: P 2ac 2ab	Cell parameters from 25 reflections
*a* = 8.5680 (17) Å	θ = 9–13°
*b* = 11.152 (2) Å	µ = 0.32 mm^−^^1^
*c* = 14.232 (3) Å	*T* = 294 K
*V* = 1359.9 (5) Å^3^	Block, colorless
*Z* = 4	0.30 × 0.20 × 0.10 mm

### Data collection

**Table:**

Enraf–Nonius CAD-4 diffractometer	1775 reflections with *I* > 2σ(*I*)
Radiation source: fine-focus sealed tube	*R*_int_ = 0.034
graphite	θ_max_ = 25.4°, θ_min_ = 2.3°
ω/2θ scans	*h* = −10→0
Absorption correction: ψ scan (North *et al.*, 1968)	*k* = −13→13
*T*_min_ = 0.910, *T*_max_ = 0.969	*l* = 0→17
2733 measured reflections	3 standard reflections every 120 min
2465 independent reflections	intensity decay: 1%

### Refinement

**Table:**

Refinement on *F*^2^	Secondary atom site location: difference Fourier map
Least-squares matrix: full	Hydrogen site location: inferred from neighbouring sites
*R*[*F*^2^ > 2σ(*F*^2^)] = 0.066	H-atom parameters constrained
*wR*(*F*^2^) = 0.188	*w* = 1/[σ^2^(*F*_o_^2^) + (0.1*P*)^2^ + 0.77*P*] where *P* = (*F*_o_^2^ + 2*F*_c_^2^)/3
*S* = 1.00	(Δ/σ)_max_ < 0.001
2465 reflections	Δρ_max_ = 0.31 e Å^−^^3^
190 parameters	Δρ_min_ = −0.33 e Å^−^^3^
0 restraints	Absolute structure: Flack (1983), 1012 Friedel pairs
Primary atom site location: structure-invariant direct methods	Flack parameter: −0.1 (2)

### Special details

**Table:**

Geometry. All e.s.d.'s (except the e.s.d. in the dihedral angle between two l.s. planes) are estimated using the full covariance matrix. The cell e.s.d.'s are taken into account individually in the estimation of e.s.d.'s in distances, angles and torsion angles; correlations between e.s.d.'s in cell parameters are only used when they are defined by crystal symmetry. An approximate (isotropic) treatment of cell e.s.d.'s is used for estimating e.s.d.'s involving l.s. planes.
Refinement. Refinement of *F*^2^ against ALL reflections. The weighted *R*-factor *wR* and goodness of fit *S* are based on *F*^2^, conventional *R*-factors *R* are based on *F*, with *F* set to zero for negative *F*^2^. The threshold expression of *F*^2^ > σ(*F*^2^) is used only for calculating *R*-factors(gt) *etc*. and is not relevant to the choice of reflections for refinement. *R*-factors based on *F*^2^ are statistically about twice as large as those based on *F*, and *R*- factors based on ALL data will be even larger.

### Fractional atomic coordinates and isotropic or equivalent isotropic displacement parameters (Å^2^)

**Table:**

	*x*	*y*	*z*	*U*_iso_*/*U*_eq_	
Cl	0.0048 (2)	0.53814 (16)	0.89390 (11)	0.0988 (6)	
O1	−0.2368 (5)	0.1837 (4)	0.4272 (2)	0.0826 (12)	
O2	−0.0472 (4)	0.3226 (3)	0.4484 (2)	0.0666 (9)	
N	−0.2332 (4)	0.2838 (4)	0.5598 (2)	0.0591 (10)	
H0A	−0.3230	0.2503	0.5658	0.071*	
F1	−0.0639 (5)	0.2695 (4)	0.9446 (2)	0.1080 (13)	
F2	−0.2866 (6)	0.2112 (4)	0.8948 (2)	0.1123 (14)	
F3	−0.2646 (5)	0.3817 (4)	0.9590 (2)	0.1006 (12)	
C1	−0.1803 (11)	0.1852 (9)	0.1733 (4)	0.113 (3)	
H1A	−0.2077	0.2306	0.1210	0.135*	
C2	−0.2231 (8)	0.2230 (6)	0.2625 (4)	0.0855 (17)	
H2A	−0.2801	0.2933	0.2705	0.103*	
C3	−0.1805 (6)	0.1559 (5)	0.3380 (3)	0.0640 (12)	
C4	−0.0945 (7)	0.0528 (6)	0.3267 (5)	0.0858 (17)	
H4A	−0.0635	0.0080	0.3785	0.103*	
C5	−0.0551 (8)	0.0171 (7)	0.2366 (7)	0.108 (2)	
H5A	0.0023	−0.0528	0.2279	0.129*	
C6	−0.0982 (10)	0.0814 (9)	0.1623 (6)	0.107 (3)	
H6A	−0.0721	0.0554	0.1023	0.128*	
C7	−0.1599 (6)	0.2711 (5)	0.4764 (3)	0.0592 (12)	
C8	−0.1749 (5)	0.3473 (4)	0.6376 (3)	0.0552 (10)	
C9	−0.0823 (6)	0.4470 (4)	0.6301 (3)	0.0630 (12)	
H9A	−0.0551	0.4759	0.5710	0.076*	
C10	−0.0296 (6)	0.5046 (5)	0.7093 (4)	0.0701 (14)	
H10A	0.0317	0.5731	0.7037	0.084*	
C11	−0.0673 (6)	0.4609 (5)	0.7972 (3)	0.0668 (13)	
C12	−0.1575 (5)	0.3606 (5)	0.8067 (3)	0.0575 (11)	
C13	−0.2113 (6)	0.3025 (4)	0.7261 (3)	0.0575 (11)	
H13A	−0.2717	0.2336	0.7316	0.069*	
C14	−0.1934 (7)	0.3082 (5)	0.9005 (4)	0.0750 (14)	

### Atomic displacement parameters (Å^2^)

**Table:**

	*U*^11^	*U*^22^	*U*^33^	*U*^12^	*U*^13^	*U*^23^
Cl	0.1108 (13)	0.0951 (10)	0.0905 (9)	−0.0134 (10)	−0.0136 (9)	−0.0296 (8)
O1	0.076 (2)	0.100 (3)	0.071 (2)	−0.028 (2)	0.0213 (18)	−0.0225 (19)
O2	0.0450 (19)	0.081 (2)	0.0734 (19)	−0.0056 (17)	0.0076 (16)	0.0014 (17)
N	0.041 (2)	0.078 (2)	0.0584 (19)	−0.0034 (19)	0.0061 (17)	−0.0029 (18)
F1	0.112 (3)	0.130 (3)	0.0824 (19)	0.030 (3)	−0.007 (2)	0.027 (2)
F2	0.146 (4)	0.112 (3)	0.0788 (19)	−0.032 (3)	0.025 (2)	0.0049 (19)
F3	0.106 (3)	0.121 (3)	0.0750 (18)	0.010 (2)	0.0225 (19)	−0.0228 (19)
C1	0.120 (6)	0.150 (8)	0.068 (3)	−0.029 (6)	0.003 (4)	0.008 (4)
C2	0.085 (4)	0.088 (4)	0.083 (3)	0.015 (4)	0.005 (3)	0.004 (3)
C3	0.055 (3)	0.078 (3)	0.060 (2)	−0.010 (3)	0.008 (2)	−0.011 (2)
C4	0.068 (4)	0.086 (4)	0.104 (4)	−0.001 (3)	−0.006 (3)	−0.004 (4)
C5	0.078 (4)	0.099 (5)	0.146 (7)	0.000 (4)	0.020 (5)	−0.052 (5)
C6	0.091 (5)	0.131 (7)	0.099 (5)	−0.021 (5)	0.032 (4)	−0.042 (5)
C7	0.045 (3)	0.074 (3)	0.059 (2)	0.011 (3)	−0.003 (2)	−0.004 (2)
C8	0.038 (2)	0.067 (3)	0.060 (2)	0.009 (2)	0.0014 (19)	0.002 (2)
C9	0.062 (3)	0.059 (3)	0.068 (3)	0.001 (2)	0.004 (2)	0.006 (2)
C10	0.069 (3)	0.063 (3)	0.079 (3)	−0.007 (3)	0.005 (3)	−0.005 (2)
C11	0.057 (3)	0.073 (3)	0.071 (3)	0.006 (3)	−0.003 (2)	−0.004 (2)
C12	0.048 (3)	0.064 (3)	0.061 (2)	0.007 (2)	0.000 (2)	−0.002 (2)
C13	0.049 (3)	0.057 (3)	0.066 (2)	0.000 (2)	0.001 (2)	0.004 (2)
C14	0.075 (4)	0.085 (4)	0.065 (3)	0.000 (3)	0.001 (3)	0.001 (3)

### Geometric parameters (Å, °)

**Table:**

Cl—C11	1.738 (5)	C4—C5	1.384 (10)
O1—C7	1.369 (6)	C4—H4A	0.9300
O1—C3	1.393 (6)	C5—C6	1.330 (12)
O2—C7	1.192 (6)	C5—H5A	0.9300
N—C7	1.350 (6)	C6—H6A	0.9300
N—C8	1.406 (6)	C8—C9	1.370 (7)
N—H0A	0.8600	C8—C13	1.390 (6)
F1—C14	1.346 (7)	C9—C10	1.373 (7)
F2—C14	1.347 (7)	C9—H9A	0.9300
F3—C14	1.318 (6)	C10—C11	1.381 (7)
C1—C6	1.363 (12)	C10—H10A	0.9300
C1—C2	1.386 (9)	C11—C12	1.366 (8)
C1—H1A	0.9300	C12—C13	1.395 (7)
C2—C3	1.360 (8)	C12—C14	1.490 (7)
C2—H2A	0.9300	C13—H13A	0.9300
C3—C4	1.375 (9)		
			
C7—O1—C3	117.2 (4)	C9—C8—C13	119.5 (4)
C7—N—C8	125.5 (4)	C9—C8—N	123.5 (4)
C7—N—H0A	117.3	C13—C8—N	116.9 (4)
C8—N—H0A	117.3	C8—C9—C10	120.4 (4)
C6—C1—C2	120.0 (7)	C8—C9—H9A	119.8
C6—C1—H1A	120.0	C10—C9—H9A	119.8
C2—C1—H1A	120.0	C9—C10—C11	120.1 (5)
C3—C2—C1	119.0 (7)	C9—C10—H10A	120.0
C3—C2—H2A	120.5	C11—C10—H10A	120.0
C1—C2—H2A	120.5	C12—C11—C10	120.7 (5)
C2—C3—C4	120.7 (5)	C12—C11—Cl	121.9 (4)
C2—C3—O1	120.3 (5)	C10—C11—Cl	117.3 (4)
C4—C3—O1	118.6 (5)	C11—C12—C13	119.1 (4)
C3—C4—C5	118.7 (6)	C11—C12—C14	121.7 (5)
C3—C4—H4A	120.6	C13—C12—C14	119.1 (5)
C5—C4—H4A	120.6	C8—C13—C12	120.2 (4)
C6—C5—C4	120.9 (7)	C8—C13—H13A	119.9
C6—C5—H5A	119.6	C12—C13—H13A	119.9
C4—C5—H5A	119.6	F3—C14—F1	106.6 (5)
C5—C6—C1	120.7 (7)	F3—C14—F2	105.3 (5)
C5—C6—H6A	119.7	F1—C14—F2	105.0 (5)
C1—C6—H6A	119.7	F3—C14—C12	114.7 (5)
O2—C7—N	128.4 (5)	F1—C14—C12	111.9 (5)
O2—C7—O1	124.2 (4)	F2—C14—C12	112.5 (4)
N—C7—O1	107.5 (4)		
			
C6—C1—C2—C3	0.7 (11)	C8—C9—C10—C11	1.2 (8)
C1—C2—C3—C4	0.9 (10)	C9—C10—C11—C12	−0.2 (8)
C1—C2—C3—O1	−172.5 (6)	C9—C10—C11—Cl	179.6 (4)
C7—O1—C3—C2	−83.3 (7)	C10—C11—C12—C13	0.0 (7)
C7—O1—C3—C4	103.2 (6)	Cl—C11—C12—C13	−179.8 (4)
C2—C3—C4—C5	−1.5 (9)	C10—C11—C12—C14	177.0 (5)
O1—C3—C4—C5	172.0 (5)	Cl—C11—C12—C14	−2.8 (7)
C3—C4—C5—C6	0.5 (10)	C9—C8—C13—C12	1.8 (7)
C4—C5—C6—C1	1.0 (12)	N—C8—C13—C12	179.9 (4)
C2—C1—C6—C5	−1.6 (12)	C11—C12—C13—C8	−0.8 (7)
C8—N—C7—O2	−11.4 (8)	C14—C12—C13—C8	−177.8 (4)
C8—N—C7—O1	167.3 (4)	C11—C12—C14—F3	58.4 (7)
C3—O1—C7—O2	−1.4 (7)	C13—C12—C14—F3	−124.6 (5)
C3—O1—C7—N	179.8 (4)	C11—C12—C14—F1	−63.3 (7)
C7—N—C8—C9	32.4 (7)	C13—C12—C14—F1	113.7 (5)
C7—N—C8—C13	−145.7 (5)	C11—C12—C14—F2	178.7 (5)
C13—C8—C9—C10	−2.0 (7)	C13—C12—C14—F2	−4.4 (7)
N—C8—C9—C10	180.0 (5)		

### Hydrogen-bond geometry (Å, °)

**Table:**

*D*—H···*A*	*D*—H	H···*A*	*D*···*A*	*D*—H···*A*
N—H0A···O2^i^	0.86	2.10	2.943 (5)	168
C9—H9A···O2	0.93	2.44	2.950 (5)	114
C13—H13A···F2	0.93	2.34	2.687 (5)	102

Symmetry codes: (i) *x*−1/2, −*y*+1/2, −*z*+1.
